# Combining a rhesus cytomegalovirus/SIV vaccine with a neutralizing antibody to protect against SIV challenges in rhesus macaques

**DOI:** 10.3389/fmicb.2025.1592647

**Published:** 2025-06-02

**Authors:** Jessica Coppola, Mara Parren, Raiza Bastidas, Karen Saye, Jacqueline Malvin, Joseph G. Jardine, Roxanne M. Gilbride, Sohita Ojha, Shana Feltham, David Morrow, Aaron Barber-Axthelm, Rachele Bochart, Randy Fast, Kelli Oswald, Rebecca Shoemaker, Jeffrey D. Lifson, Louis J. Picker, Dennis R. Burton, Scott G. Hansen

**Affiliations:** ^1^Department of Immunology and Microbiology, Scripps Consortium for HIV/AIDS Vaccine Development (CHAVD), The Scripps Research Institute, IAVI Neutralizing Antibody Center, La Jolla, CA, United States; ^2^Vaccine & Gene Therapy Institute and Oregon National Primate Research Center, Oregon Health & Science University, Beaverton, OR, United States; ^3^AIDS and Cancer Virus Program, Frederick National Laboratory, Leidos Biomedical Research, Inc., Frederick, MD, United States

**Keywords:** HIV, SIV, neutralizing antibodies, T cells, vaccine, non-human primate (macaque)

## Abstract

A vaccine is considered essential for controlling the HIV pandemic and ultimately eradicating AIDS. Neutralizing antibodies and MHC-E-restricted CD8+ T cells have shown the ability to protect against the simian counterpart of HIV, SIV, in rhesus macaques. In this study, we provide preliminary evidence that combining these orthogonal antiviral mechanisms can offer increased protection against SIV. Specifically, the replication arrest observed following vaccination with a rhesus cytomegalovirus (RhCMV/SIV)-based vaccine was enhanced by the presence of a passively administered neutralizing antibody at incompletely protective levels. This report encourages studies involving larger cohorts of macaques and alternative methods for administering neutralizing antibodies.

## Introduction

Only two vaccination modalities have consistently shown to provide protection against the human immunodeficiency virus (HIV) and/or its simian counterpart, the simian immunodeficiency virus (SIV). The first modality is neutralizing antibodies (nAbs). Autologous nAbs induced through vaccination with a stabilized recombinant envelope (Env) molecule have been demonstrated to provide protection against challenges with the corresponding HIV/SIV chimera (SHIV) in rhesus macaques (RMs) (Pauthner et al., 2017; Petitdemange et al., 2019). Furthermore, passively administered broadly neutralizing antibodies have been shown to provide protection against SHIV and SIV in RMs (Parren et al., 2001; Pegu et al., 2019; Zhao et al., 2022) and against exposure to antibody-sensitive strains of HIV in humans (Corey et al., 2021). The second modality is the MHC-E-restricted CD8+ T cell-targeted rhesus cytomegalovirus (RhCMV/SIV)-based vaccine. When properly genetically programmed (strain 68-1 and certain derivatives), RhCMV/SIV vaccine vectors elicit MHC-E-restricted CD8+ T cells in RMs, and these responses mediate the early complete arrest of SIVmac239 replication in approximately 60% of RM after they are SIV infected by repeated, limiting-dose SIV challenge, with the vast majority of these protected RMs subsequently clearing SIV infection completely (Hansen et al., 2011; Hansen et al., 2013a; Hansen et al., 2013b; Hansen et al., 2016; Hansen et al., 2019; Malouli et al., 2021; Verweij et al., 2021; Picker et al., 2023). RhCMV/SIV vaccines (typically containing Gag, Rev/Tat/Nef, and 5’-Pol inserts) do not induce antibodies and are effective in the absence of Env expression (Hansen et al., 2011; Hansen et al., 2013a; Hansen et al., 2019; Malouli et al., 2021; Hansen et al., 2022). Therefore, they represent a mode of protection completely orthogonal relative to nAbs (Hansen et al., 2011; Hansen et al., 2013a; Hansen et al., 2019; Malouli et al., 2021; Hansen et al., 2022). The MHC-E restricted CD8+ T cell responses are long-lived, with protective efficacy shown up to 10 years post-primary vaccination in RMs (Picker et al., 2023).

An important distinction exists between the nature of protection mediated by nAbs and the MHC-E-restricted CD8+ T cell responses described above. For nAbs, protection is generally observed as an early sterilizing type of immunity, with very little or no viral replication or spread observed following challenge (Hessell et al., 2007; Hessell et al., 2010; Barouch et al., 2013; Burton, 2023; Stab et al., 2023). For RhCMV/SIV-induced MHC-E restricted CD8+ T cell-mediated protection in RMs, some SIV replication and spread occur early after effective challenge, as evidenced by direct viral measurements in tissues in the first few weeks following infection and by the post-challenge induction of T cell responses to SIV antigens (Ags) not included in the vaccine (e.g., anti-Vif and anti-Env T cell responses) (Hansen et al., 2013a; Picker et al., 2023). Infection may also result in “blips” in plasma virus, and can be definitively demonstrated by the adoptive transfer of hematolymphoid cells from protected RM to naive RMs, resulting in typical SIV infection in the recipient animals (Hansen et al., 2013a; Hansen et al., 2019). In RhCMV/SIV-vaccine-protected RMs, this early stage of infection is strictly controlled (“replication arrest”), and eventually, SIV is cleared (Hansen et al., 2011; Hansen et al., 2013a; Hansen et al., 2019; Picker et al., 2023). Thus, while nAbs act to prevent viral entry and establishment of infection, RhCMV/SIV-induced T cells arrest viral replication after infection has been initiated. These fundamentally distinct mechanisms suggest the potential for complementary and possibly synergistic protection when both are present. This potential synergy arises because nAbs and MHC-E-restricted T cells act most effectively at different points along the infection timeline: nAbs reduce the initial number of infected cells, while CD8+ T cells target those that escape early antibody control. By lowering the initial viral burden, sub-protective nAbs may reduce the number of infected cells, increasing the efficiency of replication arrest and effectively lowering the immune threshold needed for successful T cell-mediated control.

Both of these effective protective modalities have significant limitations. The serum nAb titers required for sterilizing immunity against (S)HIV (and indeed against many viruses) are high, typically reaching several hundred (Pauthner et al., 2019; Pegu et al., 2019; Saunders et al., 2022; Zhao et al., 2022), and these titers are challenging to induce and sustain by vaccination. Although the RhCMV/SIV vaccine is very durable, it still only protects approximately 60% of SIV-challenged RMs (Hansen et al., 2011; Hansen et al., 2013a; Hansen et al., 2019; Picker et al., 2023). A key question is whether these two modalities can synergize, particularly when nAb titers decrease below the level required to fully prevent the infection. Specifically, does the presence of nAbs at sub-completely protective (sub-threshold) neutralizing titers at the time of challenge increase the proportion of RhCMV/SIV-vaccinated RMs undergoing replication arrest? In other words, can lower nAb titers contribute to protection in the presence of an MHC-E-restricted T-cell response to HIV?

By administering sub-protective doses of nAbs, we aimed to test whether early partial restriction of viral spread could shift outcomes in RhCMV/SIV-vaccinated animals from uncontrolled infection to replication arrest, thereby probing the threshold for effective T cell-mediated protection. We investigated these questions in a pilot study using the RM model, which utilized a 68-1 RhCMV/SIV vaccine, passive administration of an anti-SIV nAb, and SIVmac239 challenge. Passive antibody administration was selected because no SIV Env-targeted vaccine has yet been developed that can reliably induce nAbs against SIVmac239. We first identified the serum neutralizing antibody titer required for complete protection against SIVmac239 to intentionally use a sub-protective dose in the synergy experiment. We then vaccinated two cohorts of RMs with the RhCMV/SIV vaccine and 73 weeks later, they were administered nAbs or control Abs, followed by a challenge with high-dose SIVmac239. A high-dose SIV challenge was used to limit the number of passive antibody administrations required. The results from this pilot study are promising, indicating potential synergy between nAbs and MHC-E-restricted CD8+ T cells in resisting SIV infection, even under conditions of a stringent high-dose viral challenge.

## Materials and methods

### Rhesus macaques

**Figure 1:**
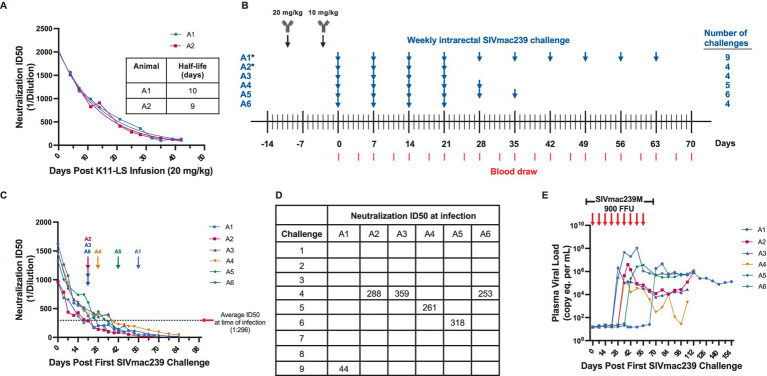
A neutralization ID50 of 1:300 is the threshold for protection against high-dose (900 FFU) SIVmac239 challenge by antibody K11-LS. **(A)** The half-life of K11-LS was determined to be 9.5 days after two RMs (A1 and A2) were administered 20 mg/kg and titers were allowed to decay. **(B)** RMs A3-A6 were administered 20 mg/kg K11-LS on day −10 followed by 10 mg/kg on day −3. *Animals A1 and A2 were administered 20 mg/kg K11-LS and titers were allowed to decay to 1:100 or less prior to a second infusion of 10 mg/kg at day 42 to calculate half-life. All RMs were challenged 3 days after the second K11-LS infusion and every week thereafter until infected (blue arrow). Red lines indicate blood draws. **(C)** Neutralization ID50s against SIVmac239 PSV were on average 1:296 at time of infection. This excludes outlier animal A1, who became infected after 9 challenges with an ID50 of 1:44, thus, exhibiting an inherent resistance to SIVmac239. Arrows indicate the last challenge before infection. **(D)** Neutralization ID50 at time of infection for each animal. **(E)** Viral load in all 6 RMs shown in panel C post first effective 900 FFU SIVmac239 challenge. Arrows indicate weekly 900 FFU challenge.

**Figure 2:**
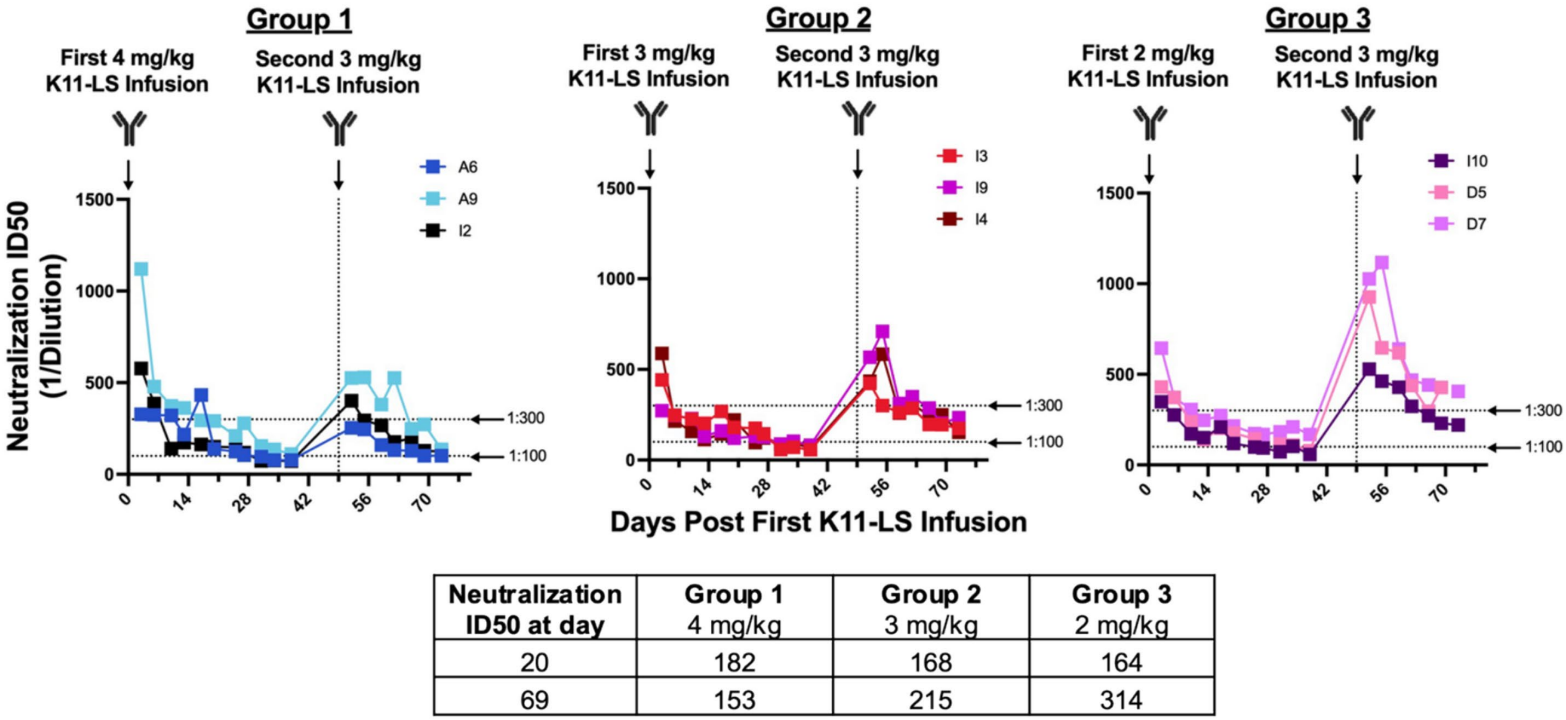
Administering K11-LS at a dose of 3 mg/kg achieves a neutralization titer between 1:100 and 1:300 within 21 days. RMs were administered 4 mg/kg (Group 1), 3 mg/kg (Group 2), or 2 mg/kg (Group 3) on day 0. 20 days later, the geometric mean ID50s were within the desired range of 1:100–1:200. A second dose of 3 mg/kg K11-LS was administered to all 3 groups on day 49 as the 3 mg/kg group exhibited the least variability between animals. 20 days after the second K11-LS infusion, geometric mean ID50s were 1:153, 1:215, and 1:314 for Groups 1, 2, and 3, respectively. A dose of 3 mg/kg of K11-LS was chosen for subsequent studies as ID50s were within the desired range and were the most consistent between animals.

**Figure 3:**
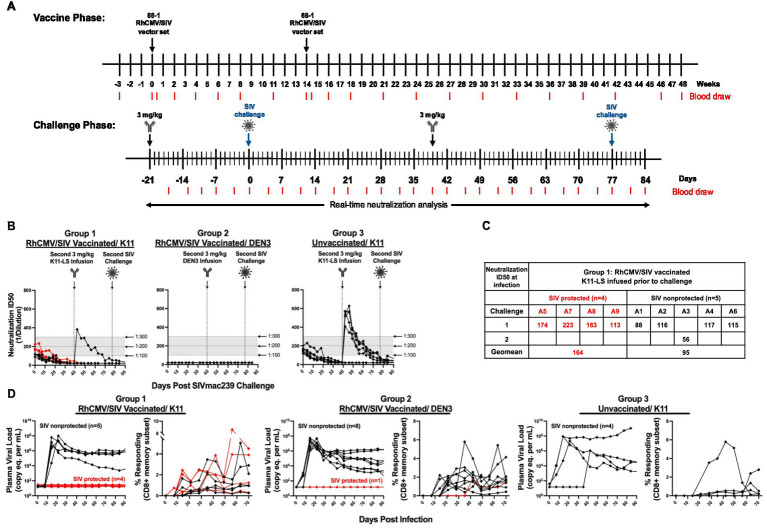
Durability and induction of both SIV-specific T cell responses and unconventionally restricted T cells elicited by 68-1 RhCMV/SIV vaccine vectors. **(A)** Longitudinal analysis of the overall SIV-specific CD4+ and CD8+ T cell responses in peripheral blood. Responses were determined by ICS analysis (TNF vs. IFN-*γ*) using whole open reading frame (ORF) mixes of overlapping 15mer peptides (Gag; Rev./Nef/Tat; 5’-Pol) to stimulate PBMC. The frequency of IFN-γ and/or TNF-positive memory T cell responses to each ORF peptide mix was summed to get the overall responses shown in the figure. Vaccinations are indicated by the arrowhead above the graph. **(B)** Longitudinal analyses of CD8+ T cell responses to individual MHC-E- (green; left panel) and MHC-II- (blue; right panel)-restricted 15mer supertopes. Responses were determined by ICS as described in **(A)**.

**Figure 4:**
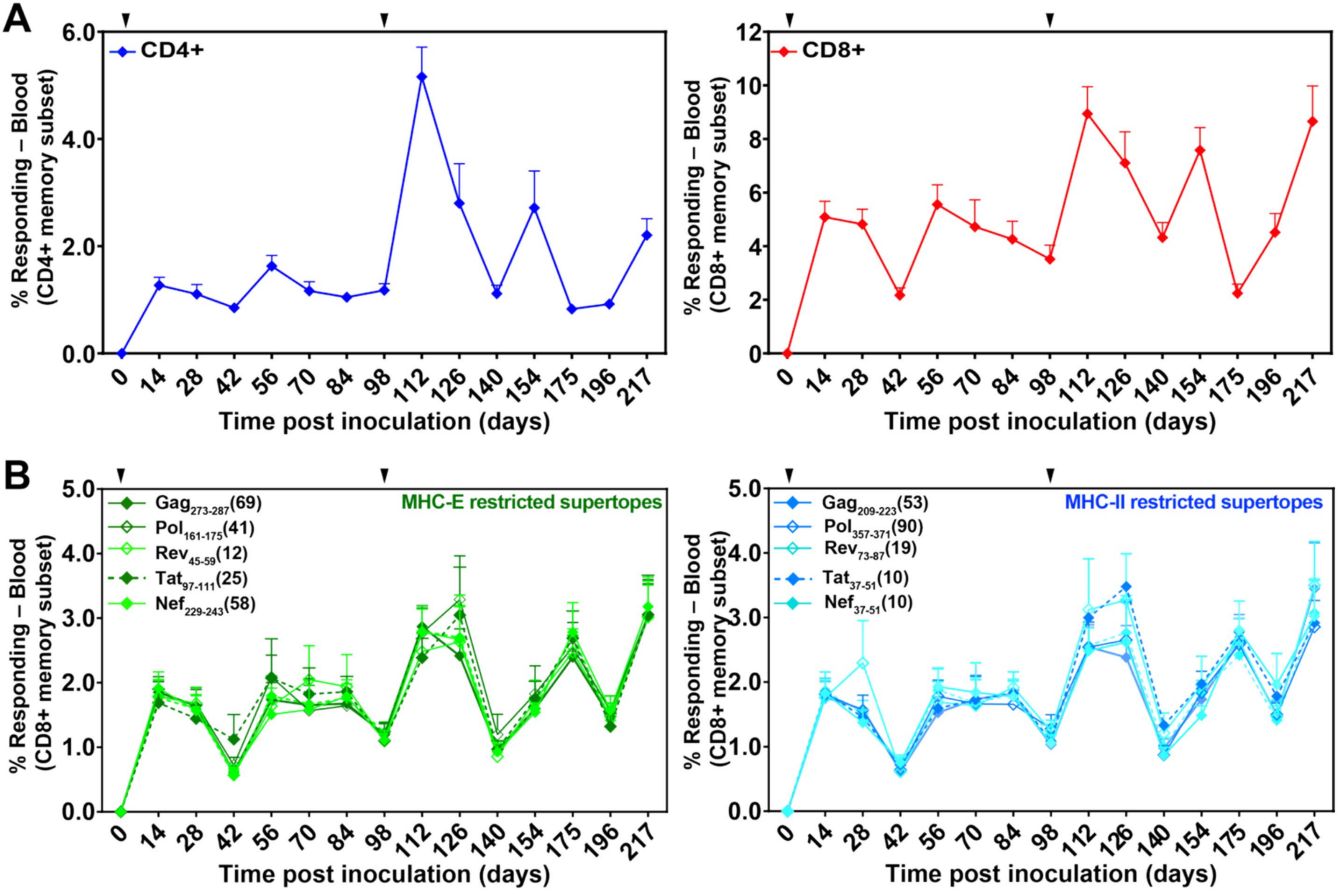
nAb K11-LS facilitates 68-1 RhCMV/SIV vector-mediated protection via replication arrest. **(A)** The vaccine phase consisted of 12 months during which RMs were administered the RhCMV/SIV vaccine on weeks 0 and 14. During the challenge phase, RMs were administered 3 mg/kg K11-LS (Groups 1 [n=9] and 3 [n=6]) or control antibody (Group 2 [n=9]) 21 to 24 days before SIVmac239 challenge. Animals A3, B3, B7, B8, C1, C3, C4, C5, and C6 received a second dose of 3 mg/kg K11-LS (Groups 1 and 3) or control antibody (Group 2) 39 days after primary challenge. RMs A3, B3, B7, B8, C3, C4, C5, and C6 were challenged a second time on day 77. RM C1, having been found to be infected after the primary challenge, was not re-challenged. **(B)** At the time of primary challenge (designated as day 0), the neutralization ID50s against SIVmac239 pseudovirus averaged 1:130 and 1:160 for Groups 1 and 3, respectively. Group 2 showed no neutralization activity, as animals received DEN3 control IgG instead of K11-LS. **(C)** Within Group 1, nAb titers at time of effective challenge were on average 1:164 and 1:95 for SIV protected and SIV non-protected RM, respectively. **(D)** Plasma viral load (PVL) and SIVmac239 Vif-specific CD8+ T cell responses are shown for infected RMs across all three groups. The x-axis represents days post effective challenge, with day 0 marking the time point at which infection was established, as indicated by the onset of plasma viremia and/or de novo detection of Vif-specific T cell responses. RMs were considered protected if they exhibited only anti-Vif responses without sustained viremia (with or without transient viral blips; shown as red lines), and non-protected if they developed sustained plasma viremia (black lines). In Group 3, four RMs exhibited sustained viremia (non-protected; black lines), while two animals showed no evidence of viral replication or Vif-specific responses and are thus not shown on the graph. No difference was observed in absolute CD4+ and CD8+ T cell levels between Groups 1 and 2 and pre- and post-vaccination.

This study used 39 Indian RMs (*Macaca mulatta*). Of these 39 RMs, 15 were used to determine the ideal conditions for the main study (Figures 1, 2), while 24 were used to evaluate the synergy between antibodies and the RhCMV/SIV vaccine (Figures 3, 4). All RMs were classified as specific pathogen-free, meaning they were free from cercopithecine herpesvirus 1, D-type simian retrovirus, simian T-lymphotropic virus type 1, SIV, and *M. tuberculosis*, although they were naturally infected with RhCMV at the start of the study. All RMs involved in this study were housed at the Oregon National Primate Research Center (ONPRC) in Animal Biosafety Level 2+ rooms. RM care and all experimental protocols and procedures were approved by the ONPRC Institutional Animal Care and Use Committee (IACUC). The ONPRC is a category I facility. The Laboratory Animal Care and Use Program at the ONPRC is fully accredited by the American Association for Accreditation of Laboratory Animal Care, and the program has an approved assurance (No. A3304-01) for the care and use of animals, which is documented with the National Institutes of Health for Protection from Research Risks. The IACUC adheres to national guidelines established by the Animal Welfare Act (7 U.S.C. Sections 2131-2159) and the Guide for the Care and Use of Laboratory Animals (eighth edition) as mandated by the US Public Health Service Policy. RMs were administered either DEN3 or K11-LS intravenously (IV) at a controlled rate by trained personnel, following the IACUC-approved protocol of the study, with no adverse events observed.

### Cell lines

TZM-bl cells (NIH AIDS Reagent Program) were used for the pseudovirus neutralization assay. Human HEK 293 T cells (ATCC) were used for pseudovirus production. Expi293F cells (ThermoFisher) were used for monoclonal antibody production.

### SIV pseudovirus production

SIV pseudovirus Env construct was co-transfected with Env-deficient backbone plasmid (pSG3ΔEnv) in a 1:2 ratio using the transfection reagent FuGENE 6 (Promega) in HEK 293 T cells, following the manufacturer’s instructions. Cells were cultured in Dulbecco’s Modified Eagle’s Medium (DMEM), which contained 4.5 g/L of glucose and sodium pyruvate and supplemented with 10% fetal bovine serum (FBS), 2 mM L-glutamine (Gibco), and 100 U/mL Penicillin–Streptomycin Solution (Gibco). The cultures were maintained at 37°C in a humidified incubator with 5% CO₂. After 72 h of transfection, the supernatants containing the viruses were harvested, sterile-filtered (0.22 μm) (EMD Millipore), and frozen at −80°C for long-term storage.

### Antibody production and characterization

Antibody heavy chain (HC) and light chain (LC) constructs were transiently expressed using the Expi293 Expression System (ThermoFisher). HC and LC plasmids were cotransfected at a 1:2.5 rasstio using the transfection reagent FectoPRO (Polyplus) in Expi293 cells, according to the manufacturer’s instructions. Cells were cultured in Expi293™ Expression Medium (Gibco), which was supplemented with 10% Opti-MEM™ I Reduced Serum Medium (Gibco) and maintained at 37°C on a shaker in a humidified incubator with 5% CO₂. After 24 h, the cell culture media were supplemented with 300 mM valproic acid and 40% glucose (Gibco). After 5 days of transfection, the cell supernatants were harvested and sterile filtered (0.22 μm). The antibody was purified by Protein A Sepharose (GE Healthcare), as described previously (Sok et al., 2013). The antibody batches were analyzed using an analytical high-performance liquid chromatography (HPLC) system (Agilent 1260 Infinity II) to ensure they were of the correct size and purity. They were also examined in neutralization assays to confirm batch-to-batch potency, and endotoxin levels were tested before pooling all batches and freezing at −80°C for long-term storage or shipment to OHSU.

### TZM-bl neutralization assay

Serially diluted heat-inactivated serum or antibodies were incubated with SIVmac239 pseudovirus or murine leukemia (MLV) (negative control) pseudovirus in half-area 96-well white plates using DMEM (Gibco) supplemented with 10% FBS, 2 mM L-glutamine (Gibco), and 100 U/mL Penicillin/Streptomycin (Gibco). K11-LS and DEN3 served as positive and negative antibody controls, respectively. After incubating at 37°C for 1 h, TZM-bl cells (with 20 μg/mL DEAE-dextran) were added onto the plates at 10,000 cells/well. Final antibody concentrations for the dilution series were calculated based on the total volume of the assay (antibody or serum + virus + TZM-bl cells). After 72 h of incubation at 37°C in a humidified incubator with 5% CO₂, culture supernatants were removed, and the cells were lysed in a luciferase lysis buffer (25 mM Gly-Gly pH 7.8, 15 mM MgSO_4_, 4 mM EGTA, 1% Triton X-100). Luciferase activity was measured by adding Bright-Glo (Promega), according to the manufacturer’s instructions. The assays were tested in duplicate wells and were independently repeated at least twice. Neutralization IC50 or ID50 titers were calculated using “One-Site Fit LogIC50” regression in GraphPad Prism 10.

### T-cell assays

Intracellular cytokine staining (ICS) was performed as described below. SIV-specific CD4+ and CD8+ T-cell responses were analyzed in peripheral blood mononuclear cells (PBMCs) using flow cytometric ICS, as previously described (Hansen et al., 2011; Hansen et al., 2013a; Malouli et al., 2021; Hansen et al., 2022). T-cell responses to total SIV antigens were measured using combinations of sequential 15-mer peptides (with an 11 amino acid overlap) that encompass the SIVmac239 Gag, Pol, Nef, Rev., Tat, and Vif proteins. Mononuclear cells were stimulated in the presence of antibodies, namely anti-CD28 (CD28.2, Purified 500 ng/test; Life Tech, CUST03277) and anti-CD49d (9F10, Purified 500 ng/test; Life Tech, CUST03278). They were then incubated in R10, RPMI supplemented with 10% newborn calf serum (Hyclone, SH30401.01) at 37°C in a humidified incubator with 5% CO₂, along with individual peptides or peptide mixtures and antibodies for 1 h, followed by an additional 8-h incubation in the presence of Brefeldin A (5 μg mL^−1^; BioLegend, 91850). Stimulation without peptides served as a background control.

After incubation, the stimulated cells were stored at 4°C until staining with combinations of fluorochrome-conjugated monoclonal antibodies including anti-CD3 (SP34-2: PacBlue, BD Biosciences, 624034), anti-CD4 (L200; BV510, BD Biosciences, 624340), anti- CD8α (SK-1: Life Technologies, CUST04424), anti-CD69 (FN50: PE/Dazzle594, BioLegend, 93437), anti-IFNγ (B27: APC, BioLegend, 96019), anti-TNFα (Mab11: PE, BioLegend, 96019), and anti-Ki67 (B57: BD Biosciences, 624046). For memory phenotyping from whole blood, the following antibodies were used: anti-CCR5 (3A9: APC, BD Biosciences, 624346), anti-CCR7 (G043H7: Biotin, BioLegend, 93747), Streptavidin (BUV496, BD Biosciences, 624283), anti-CD20 (2H7: APC-Fire 750, BioLegend, 93924), anti-CD28 (CD28.2: PE/Dazzle594, BioLegend, 93924), anti-CD3 (SP34-2, BUV395, BD Biosciences, 624310), anti-CD8β (2ST8.5H7: BUV563, BD Biosciences, 624284), anti-CD25 (2A3: BUV737, BD Biosciences, 624286), anti-CXCR5 (MU5UBEE: SuperBright436, Life Technologies, 62-9185-42), anti-CD95 (DX2: BV605, BioLegend, 93384), anti-CD69 (FN50: BV650, BioLegend, 93755), anti-CD8α (RPA-T8: BV711, BioLegend, 900006277), anti-PD-1 (eBioJ105: SuperBright780, Life Technologies, 78-2799-42), anti- *γ*δTCR (B1; PerCP-eFluor710, BioLegend, 900002746), anti-CD127 (HIL-7R-M21: PE, BD Bioscience, 624048), anti-HLA-DR (L243: PE/Dazzle 594, BioLegend, 93957), anti-CD4 (L200: BV510, BD Biosciences, 624340), and anti-Ki67 (B57: FITC, BD Biosciences, 624046).

Stained samples were analyzed on an LSR-II or FACSymphony A5 flow cytometer (BD Biosciences). Data analysis was performed using FlowJo software (BD Biosciences). In all analyses, gating on the lymphocyte population was followed by the separation of the CD3+ T-cell subset and progressive gating on CD4+ and CD8+ T-cell subsets. Antigen-responding cells in both CD4+ and CD8+ T-cell populations were determined by their intracellular expression of CD69 and one or both of the cytokines IFN-γ and TNFα. The assay limit of detection was determined at 0.05% as previously described (Hansen et al., 2011; Hansen et al., 2013a; Hansen et al., 2019; Malouli et al., 2021; Hansen et al., 2022), after background subtraction being the minimum threshold used in this study. After background subtraction, the raw response frequencies above the assay limit of detection were “memory-corrected” (e.g., % responding out of the memory population), as described (Hansen et al., 2011; Hansen et al., 2013a; Hansen et al., 2019; Malouli et al., 2021; Hansen et al., 2022). For memory phenotype analysis, CD4+ or CD8+ T cells were subdivided into the memory subsets of interest based on surface phenotype (CD28 vs. CD95), with memory defined as CD28+/− and CD95+.

### SIVmac239 challenge experiments

To define the half-life of K11-LS, two RMS were first administered with 20 mg/kg, allowing the neutralizing titers to decay, followed by a second dose of 10 mg/kg, after which the half-life was determined. A second group of four RMs was treated with 20 mg/kg K11-LS followed by 10 mg/kg of K11-LS (Figure 1). All RMs were then challenged with a dose of 900 focus-forming units (FFU) of SIVmac239. RMs were challenged weekly until a documented instance of infection, as indicated by sustained plasma viremia, was achieved. The SIVmac239 stock was titered using the CMMT-CD4-LTR-*β*-Gal sMAGI cell assay (National Institutes of Health AIDS Reagent Program). The dose of 900 FFU was selected based on titering experiments where 100% of unvaccinated, untreated animals became infected after two challenges. For the combined K11-LS/T cell protection studies, at the end of the vaccine phase, all vaccinated and unvaccinated RMs were SIV-challenged intrarectally with a dose of 900 FFU of SIVmac239 until infection could be documented as either onset of sustained plasma viremia and/or *de novo* development of CD4+ and CD8+ T cell responses to SIVvif, at which time challenge was discontinued, as previously described (Hansen et al., 2011; Hansen et al., 2013b; Hansen et al., 2019).

### Viral load measurement

Plasma SIV RNA levels were determined using an SIV Gag-targeted quantitative RT-PCR format assay, with six replicate reactions analyzed per extracted sample for an assay threshold of 15 SIV RNA copies/ml, as previously described (Bolton et al., 2016).

## Results

### Passive transfer studies of K11 to determine conditions for synergy experiment

The monoclonal nAb K11 was originally isolated from a SIVmac239-infected RM. It binds to a glycan hole on gp120 and neutralizes SIVmac239 with an IC50 of 100 ng/mL (Zhao et al., 2022). This high potency makes it a valuable tool for investigating potential synergy with other immune responses. A half-life extended version (Ko et al., 2014) of K11, known as K11-LS, was generated, and its half-life was determined to be approximately 9.5 days in RMs, as assessed from neutralization ID50s against the SIVmac239 pseudovirus (PSV), following a single infusion of 20 mg/kg of K11-LS (Figure 1A). In a preliminary study, RMs were administered 20 mg/kg of K11-LS, allowing titers to decay to 1:100 or lower before administering a second dose of 10 mg/kg, followed by weekly intrarectal challenge with 900 FFU SIVmac239M (Fennessey et al., 2017; Khanal et al., 2019) (Figure 1B). The study indicated that maintaining a neutralization ID50 above 1:300 prevented SIVmac239 infection in most RMs (Figures 1C–E). Notably, animal A1 remained uninfected until after nine challenges despite having an ID50 of just 1:44. While including this outlier would suggest a more conservative protective threshold of 1:250, excluding it suggests a more generalizable threshold of 1:300. Accordingly, to assess synergy with the RhCMV/SIV vaccine, nAb titers were required to fall below 1:300 prior to SIVmac239 challenge.

Notably, this protective threshold differs to some degree from the previously reported ID50 of 1:609 necessary for protection when RMs were challenged intravenously with low-dose SIVmac239 (Zhao et al., 2022). This may reflect the use of a different challenge dose and/or different challenge routes in the two studies. To achieve a neutralization ID50 of 1:200 within 21 days, different doses of K11-LS (4, 3, and 2 mg/kg) were administered to nine RMs, with neutralization titers determined twice weekly. A dose of 3 mg/kg was identified as optimal for achieving the desired ID50 within the given time period (Figure 2).

### The frequency of replication arrest efficacy was higher with the combined RhCMV/SIV vaccine and passive suboptimal neutralizing antibody than with the vaccine alone

In the next study, to investigate the potential ability of incompletely protective levels of nAb to enhance protection when combined with RhCMV/SIV vaccination, 24 RMs were divided into two groups of nine each and a control group of six. Groups 1 and 2 received RhCMV/SIV vaccination, and Group 3 remained unvaccinated. Vaccination was carried out over 12 months during which RMs were administered the RhCMV/SIV vaccine twice, on weeks 0 and 14, as previously described (Hansen et al., 2011; Hansen et al., 2013a; Hansen et al., 2019). The induction of SIV-specific CD4+ and CD8+ was monitored longitudinally in blood, as shown in Figure 3A. The analysis included CD8+ T-cell responses to individual MHC-E and MHC-II-restricted 15mer supertopes (Figure 3B).

Twelve months after the first vaccination, Groups 1 and 3 received 3 mg/kg of K11-LS, while Group 2 received 3 mg/kg of DEN3, a Dengue virus control antibody, 21–24 days before high-dose (900 FFU) SIVmac239 challenge (Figures 4A,B). At the time of this primary challenge, neutralization ID50s for Groups 1 and 3 were within the desired range of 1:100–1:200 (specifically, geomean values of 1:131 and 1:153, respectively), while Group 2 exhibited no neutralization activity (Figure 4B). Following the challenge, one out of nine RMs in Group 1, two out of nine RMs in Group 2, and four out of six RMs in Group 3 remained uninfected, as evidenced by the absence of PVL and anti-Vif T-cell responses. The neutralization titers of the uninfected animals in Groups 1 and 3 were allowed to decrease to undetectable levels before a second dose of 3 mg/kg K11-LS or control antibody (Group 2) was administered on day 39. These RMs were then subsequently rechallenged on day 77, after their neutralization titers had reduced below 1:200 (Figures 4A,B).

After rechallenge, all the RMs in Groups 1 and 2 became infected as indicated by the development of Vif-specific T-cell responses (Figure 4D). In Group 1 (RhCMV/SIV + K11-LS), four out of nine animals (44%) exhibited typical replication arrest as characterized by undetectable levels of virus in plasma (Figure 4D). In Group 2 (RhCMV/SIV + DEN3), only one out of nine animals showed replication arrest; the remaining RMs exhibited robust SIV replication (Figure 3D). Notably, the frequency of replication arrest in Group 2 (11%) was significantly lower in response to the high-dose SIVmac239 challenge (900 FFU) compared to the 50–60% previously observed in studies using repeated limiting-dose (100 FFU) viral challenges (Hansen et al., 2011; Hansen et al., 2013a; Hansen et al., 2013b; Hansen et al., 2016; Hansen et al., 2019; Malouli et al., 2021; Verweij et al., 2021). In Group 3, four out of six animals became infected and exhibited robust replication of virus; two out of six animals were not infected and showed no indication of any viral replication as assessed by plasma viral load measurements or anti-Vif T-cell responses (Figure 3D). It appears that these two animals were completely protected by K11, despite the serum neutralizing titers being below the 1:300 threshold, or that they had some inherent levels of resistance to SIVmac239 infection. Importantly, the viral loads between groups appeared comparable by inspection, and there were no significant differences in viral load at peak or set point between the three groups. Among progressively infected RMs, the average peak plasma viral load was 3.95 × 10^7^ (Group 1), 2.46 × 10^7^ (Group 2), and 3.02 × 10^8^ (Group 3). The average viral load at set point (days 63–91) was 5.68 × 10^5^ (Group 1), 3.58 × 10^5^ (Group 2), and 1.165 × 10^8^ (Group 3).

On average, the neutralization ID50 in replication-arrested RMs in Group 1 was 1:164, whereas non-protected RMs had an ID50 of 1:95 at the time of effective challenge when treated with RhCMV/SIV and K11-LS (Figure 4C). This finding suggests that the threshold of neutralization titers necessary for a synergistic effect may be quite high.

## Discussion

We present here a pilot study examining the potential increase in protective activity against SIV infection by combining the orthogonal antiviral properties of a RhCMV/SIV vaccine and neutralizing antibodies. The number of animals involved was not sufficient to draw definitive conclusions, but the study does suggest certain trends that indicate a larger study employing more RMs is merited. With the caveat that it is a single experiment with small sample size of animals, this is the first study to assess the RhCMV/SIV vaccine efficacy against a high-dose SIVmac239 challenge and suggests that it is less effective against this challenge dose than the previously used low-dose viral challenge (Hansen et al., 2011; Hansen et al., 2013a; Hansen et al., 2019; Picker et al., 2023). Thus, replication arrest and protection were observed in only 11% of animals compared to the typically observed rate of approximately 60%, albeit in much larger cohorts of RMs to establish the latter figure. Even with this higher dose challenge, replication arrest and protection were observed in 44% of RhCMV/SIV-vaccinated animals when serum neutralizing antibody titers were in the range of 100–200, compared to 11% in the absence of neutralizing antibodies. We observed a higher rate of protection in RMs treated only with neutralizing antibodies than expected; however, the nature of protection was distinct, as it appeared to involve sterilizing immunity with no evidence of virus replication and, therefore, no replication arrest.

Notably, the data revealed variability in antibody-mediated protection, despite administering the same neutralizing antibody at the same dose and timing across animals likely due to a complex interplay of host immune factors. The kinetics and strength of the early innate response are crucial; an animal that initiates a rapid, robust, and well-regulated innate response (involving cytokines, interferons, and complement activation) shortly after infection may suppress the virus sufficiently for the effectiveness of the antibody’s sub-optimal neutralization or effector functions (Alter et al., 2020; Gorini et al., 2020; Rosen et al., 2024). Genetic variations, or polymorphisms, in human and macaque Fc gamma receptors (FcγRs) also modulate antibody-mediated protection against HIV and SIV (Cocklin and Schmitz, 2014). High-affinity or highly expressed FcγRs, influenced by these polymorphisms, enable the antibody to engage innate immune cells more effectively for viral clearance (Hessell et al., 2007). Furthermore, the functional capacity of innate effector cells, particularly NK cells, which mediate antibody-dependent cellular cytotoxicity (ADCC), also varies; an animal with highly potent NK cells might successfully clear infected cells targeted by the low-titer antibody, while another with less functional NK cells might not (Madhavi et al., 2013; Huot et al., 2021; Grunst et al., 2023). These findings underscore that innate immune variability can modulate the outcome of antibody-based interventions. In this context, combining RhCMV/SIV vaccination with bnAb delivery may buffer against such host-dependent variation, with MHC-E-restricted CD8+ T cells providing a durable, complementary mechanism of viral control.

Taken together, our observations suggest that the suboptimal nAb titers at the time of SIV challenge reduced the magnitude of the initial infection, thereby lowering the effective infectious dose. Thus, we posit that, as expected given their orthogonal mechanisms of antiviral activity, these immune modalities are potentially mutually supportive: RhCMV/SIV vaccination provides cell-mediated replication arrest-type protection when nAb titers are too low to sterilize the challenge. Furthermore, even a sub-optimal level of neutralization might lower the effective infectious dose, allowing for vaccine protection when the viral load is too high for the vaccine-induced MHC-E-restricted CD8+ T cells to arrest completely on their own.

When considering follow-up studies to this report, a primary objective would be to provide statistically significant proof of concept in RMs regarding the combined efficacy of nAbs and RhCMV/SIV-induced MHC-E-restricted CD8+ T cells to protect against SIV. Based on our power calculations, we estimate that a minimum of 32 animals per group would be required to achieve 80% statistical power. We suggest that this could be achieved in the limiting dose SIVmac239 challenge model by microdosing neutralizing antibodies, i.e., administering low doses of nAbs frequently to RhCMV/SIV-vaccinated animals. This strategy aims to maintain subprotective serum concentrations while subjecting the animals to repeated SIVmac239 challenge. An alternative approach is to provide an approximately constant level of subprotective nAbs through Adeno-Associated Virus (AAV) delivery (Johnson et al., 2009; Martinez-Navio et al., 2020) and repeatedly challenging vaccinated animals. Finally, and perhaps most desirably, a study is needed to identify vaccine constructs capable of eliciting nAbs against SIVmac239, enabling the testing of the efficacy of such putative vaccines both alone and in combination with RhCMV/SIV vaccination.

From unpublished data, immunization of RMs with the SIVmac239.K180S soluble trimer formulated with the saponin/MPLA nanoparticle (SMNP) adjuvant generated an antibody response that was non-neutralizing. This limited immunogenicity may be due to SIVmac239’s dense glycan shield, which is more extensive than that of HIV-1, potentially restricting access to conserved neutralizing epitopes (Zhao et al., 2022). Therefore, selective removal of glycans may help create points of access to elicit neutralizing antibodies. Another strategy to enhance the immunogenic potential of SIVmac239 Env is to further stabilize the trimer. BG505 SOSIP optimization offers a valuable model for such structural improvements. For example, replacing the furin cleavage site with flexible linkers, as demonstrated in the native flexibly linked (NFL) (Sarkar et al., 2018), Link14 (Willis et al., 2022), and uncleaved prefusion-optimized (UFO) designs (Kong et al., 2016), facilitates cleavage-independent expression of well-folded trimers while preserving their native antigenicity. Additional stabilization can be achieved through intra-protomer disulfide bonds that lock gp120 in the prefusion conformation and reduce spontaneous trimer opening or through structure-guided point mutations to fill hydrophobic cavities in gp120, increasing thermostability and minimizing exposure of non-neutralizing epitopes (Chuang et al., 2017). These strategies could provide useful insights for designing the next generation of SIVmac239 SOSIP.

## Data Availability

The datasets presented in this study are available in online repositories. The names of the repository/repositories and accession number(s) can be found in the article or Supplementary material.
