# Supplementary material for: Combining a rhesus cytomegalovirus/SIV vaccine with a neutralizing antibody to protect against SIV challenges in rhesus macaques
Source: Front Microbiol. 2025 Jun 2;16:1592647. doi: 10.3389/fmicb.2025.1592647 (PMC12171355; doi:10.3389/fmicb.2025.1592647)
Supplement: Supplementary file 1 [file Data_Sheet_1.pdf]

Neutralization ID50 at challenge	Group 1: RhCMV/SIV vaccinated K11-LS infused prior to challenge (n=9)									Group 3: Unvaccinated K11-LS infused prior to challenge (n=6)					
Challenge	A1	A2	A3	A4	A5	A6	A7	A8	A9	C1	C2	C3	C4	C5	C6
1	88	116	114	117	174	115	223	163	113	148	135	171	157	201	118
2			56									86	55	88	50

Supplementary Table 1. Neutralization ID50s for K11-LS infused RMs at time of challenge. RMs were only challenged a second time if they remained uninfected after the first challenge. RhCMV/SIV- mediated protected animals are indicated in red. No neutralization was seen from RMs in Group 2 as they received DEN3 control IgG instead of K11-LS (not shown).
